# Supplementary material for: Identification and characterization of a carbohydrate recognition domain-like region in Entamoeba histolytica Gal/GalNAc lectin intermediate subunit
Source: Microbiol Spectr. 2024 Oct 4;12(11):e00538-24. doi: 10.1128/spectrum.00538-24 (PMC11537071; doi:10.1128/spectrum.00538-24)
Supplement: Supplemental material — Fig S1 to S5; Table S1 to S4. [file spectrum.00538-24-s0001.pdf]

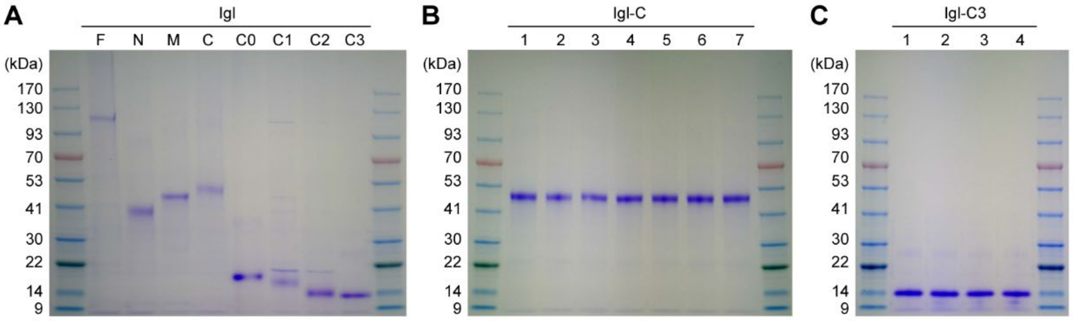

Fig. S1 Prokaryotic expression of recombinant Igl proteins. (A) Igl-F and its various segments. (B) Igl-C and its mutations. Lanes 1 to 7 are WT, C613G, C854G, C913G, C1035G, C1049G, C1068G proteins successively. (C) Igl-C3 and its mutations. In turn, lanes 1 to 4 are WT, C1035G, C1049G, C1035G/C1049G (double-point mutation) proteins. Protein purity in the figure was confirmed by SDS-PAGE and CBB staining.

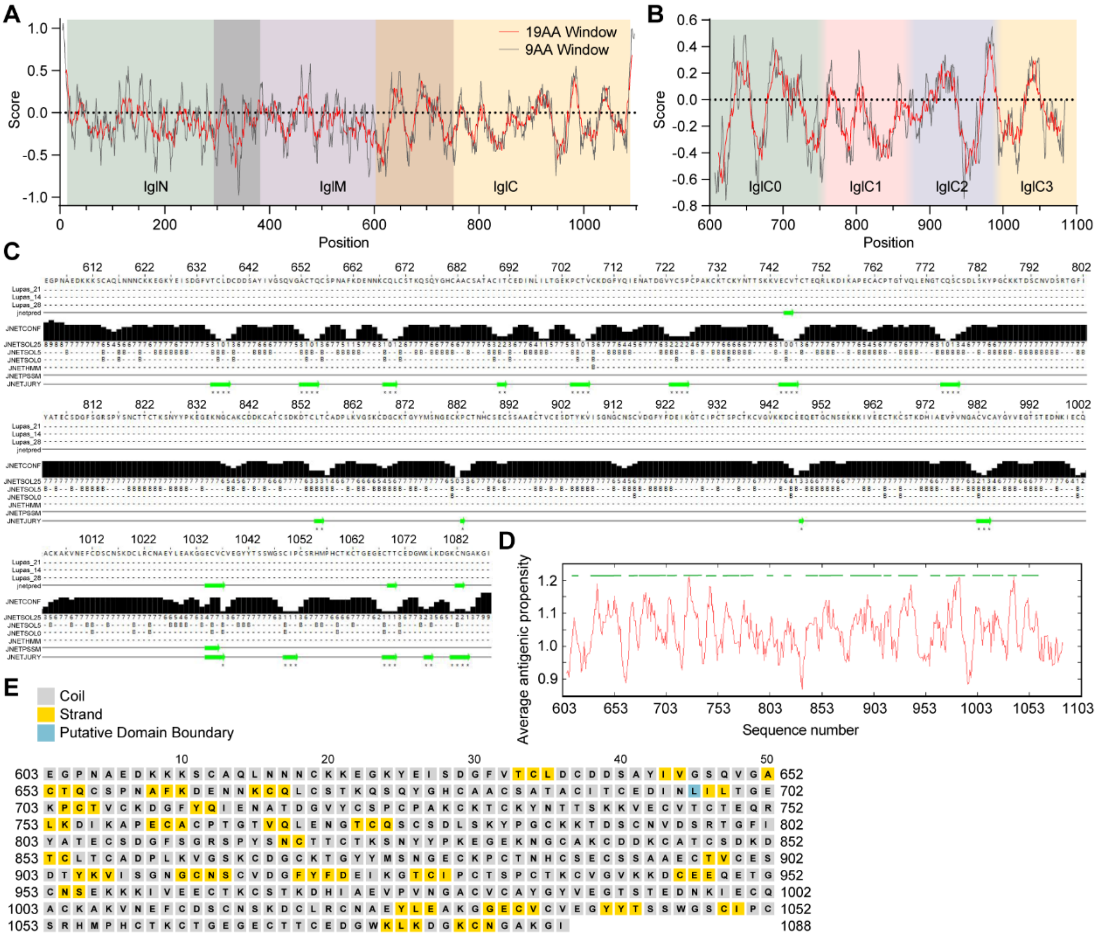

Fig. S2 Segmentation of Igl protein and prediction of its potentially key CXXC motifs. (A) Igl-F segmentation according to hydrophobicity prediction. (B) Igl-C segmentation according to hydrophobicity prediction. Hydrophobicity of Igls was calculated by Expasy ProtScale (https://web.expasy.org/protscale/) with an amino acid scale published by Hphob. / Eisenberg et al. Window size was set to 9 or 19 in a linear weight variation model. (C) Secondary structure of Igl-C predicted using Jpred 4 (https://www.compbio.dundee.ac.uk/jpred/). Jnetpred: the consensus prediction, and sheets are marked as light green arrows. JNETCONF: confidence estimates for the prediction with high values meaning high confidence. JNETHMM: the HMM profile-based prediction. JNETPSSM: the PSSM profile-based prediction, and sheets are marked as light green arrows. JNETJURY: an asterisk in this annotation indicates the rationalization of significantly different primary predictions. Jnet version: 2.3.1. (D) Antigenic peptides of Igl-C predicted using the method of Kolaskar and Tongaonkar (http://imed.med.ucm.es/Tools/antigenic.pl). Predicted antigenic determinants in the sequence are indicated by green lines. Average antigenic propensity for Igl-C protein is 1.0455. (E) Secondary structure (PSIPRED 4.0 method) and protein domain (DomPred method) prediction of Igl-C using PSIPRED (http://bioinf.cs.ucl.ac.uk/psipred/). Coil: random-coil. Strand: β-sheet.

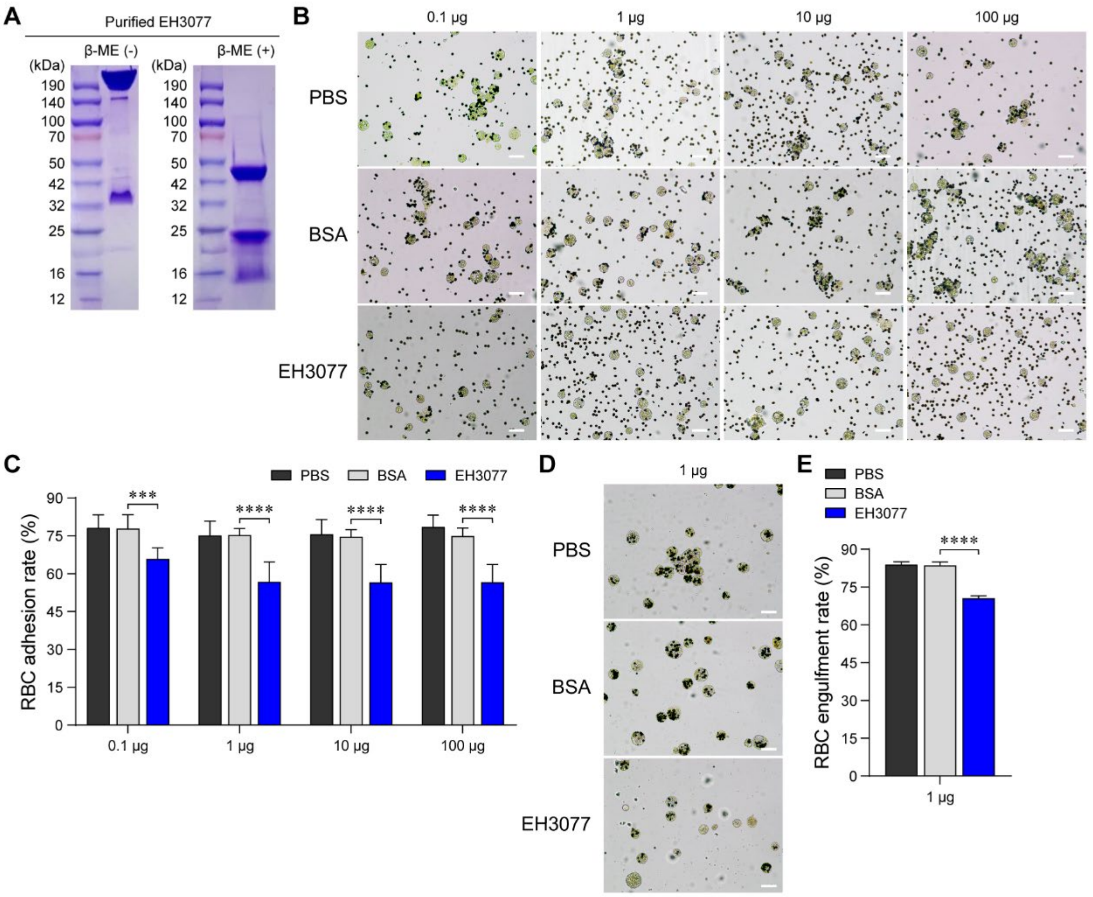

Fig. S3 Characterization of mAb EH3077 and its inhibition of trophozoite adherence and phagocytosis. (A) Band locations of non-reduced and reduced purified EH3077 confirmed by SDS-PAGE and CBB staining. (B) Erythrocytic adherence of *E. histolytica* trophozoites blocked with EH3077 and BSA. Representative images are shown. Scale bar: 100 μm. (C) Bar chart displaying statistics of RBC adhesion rate after at least three repeated experiments. (D) *E. histolytica* erythrophagocytosis blocked with EH3077 and BSA. Representative images are shown. Scale bar: 100 μm. (E) Bar chart displaying statistics of RBC engulfment rate after at least three repeated experiments. ***$p < 0.001$; ****$p < 0.0001$ by Student's t-test.

A

B

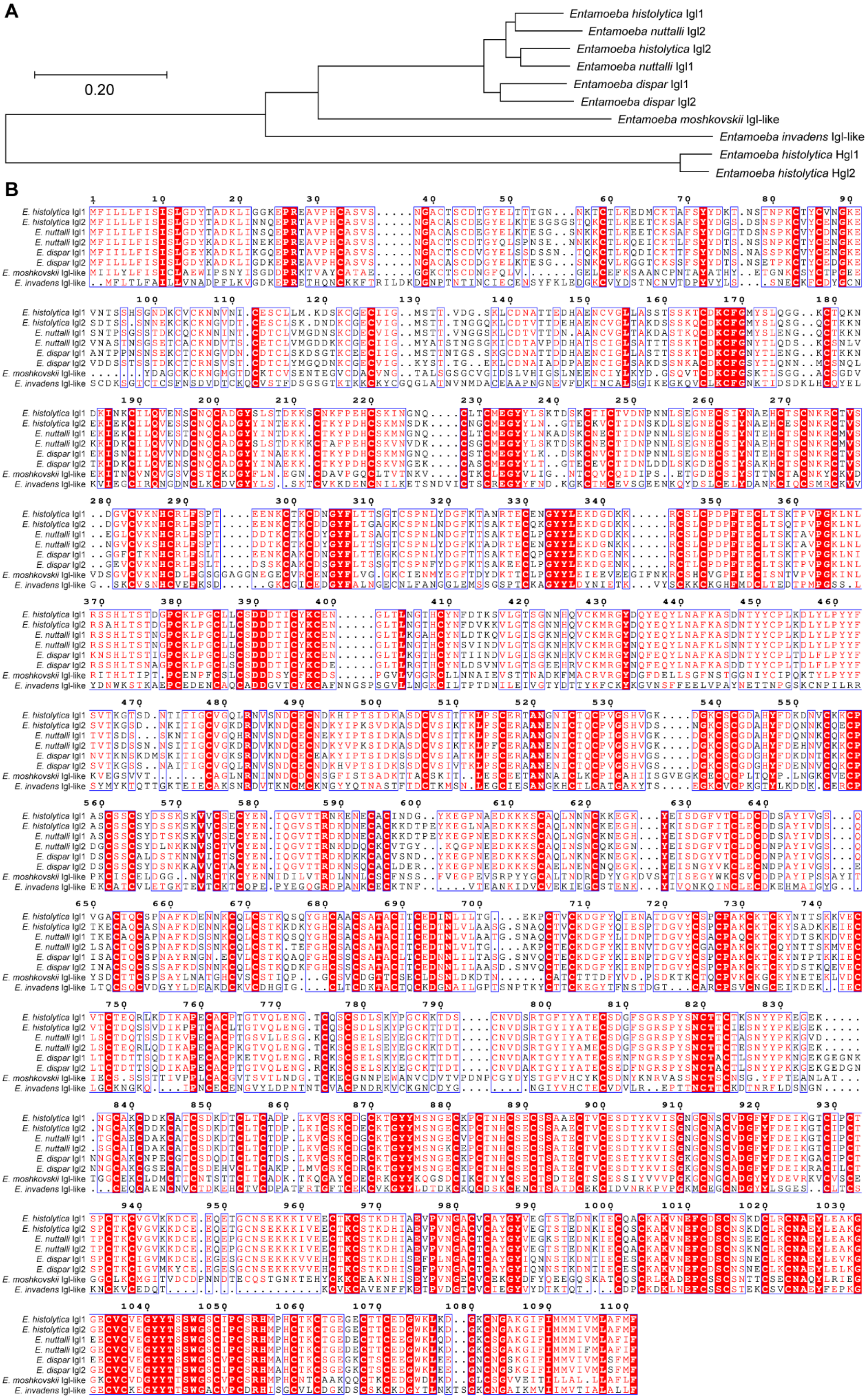

0.20
Entamoeba histolytica Igl1
Entamoeba nuttalli Igl2
Entamoeba histolytica Igl2
Entamoeba nuttalli Igl1
Entamoeba dispar Igl1
Entamoeba dispar Igl2
Entamoeba moshkovskii Igl-like
Entamoeba invadens Igl-like
Entamoeba histolytica Hgl1
Entamoeba histolytica Hgl2
E. histolytica Igl1
E. histolytica Igl2
E. nuttalli Igl1
E. nuttalli Igl2
E. dispar Igl1
E. dispar Igl2
E. moshkovskii Igl-like
E. invadens Igl-like

Fig. S4 Genetic relationship and sequence alignment of various *Entamoeba* Igls. (A) The neighbor-joining phylogenetic tree constructed using the amino acid sequences of *Entamoeba* Igls. Bar indicates 0.20 changes per amino acid position. MEGA version: 11.0.13. (B) Amino acid sequence alignment of various *Entamoeba* Igls. Alignments were produced using ClustalW and figures were generated using ESPript 3.0 (https://espript.ibcp.fr/ESPript/cgi-bin/ESPript.cgi).

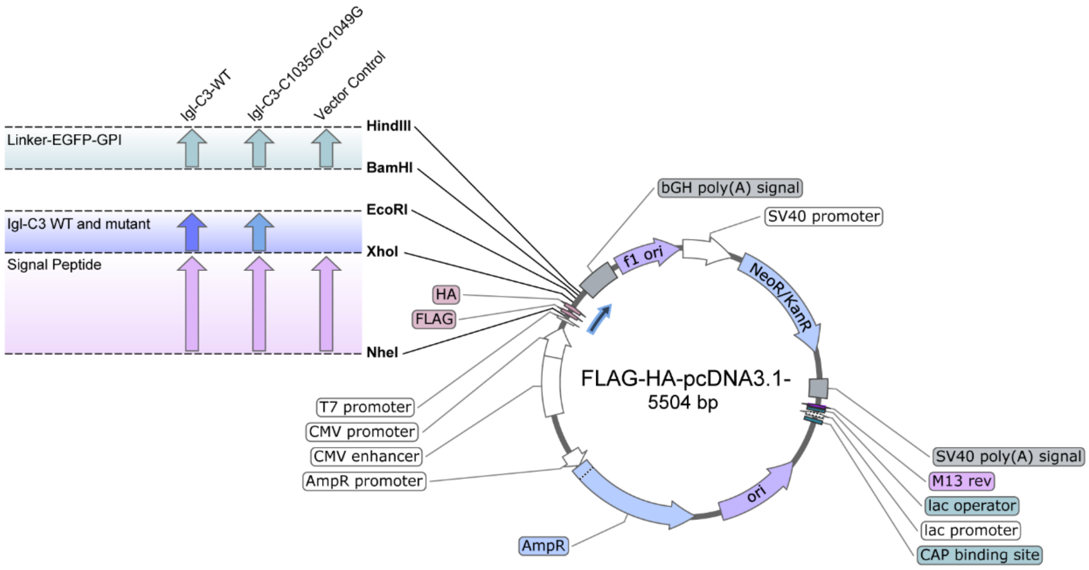

Fig. S5 Schematic diagram of recombinant FLAG-HA-pcDNA3.1- vector constructions used for expression of fusion proteins on the CHO cell surface. Signal peptide was inserted between NheI and XhoI restriction sites, linker-EGFP-GPI was inserted between BamHI and HindIII restriction sites, while Igl-C3-WT or Igl-C3-C1035G/C1049G was inserted between XhoI and EcoRI restriction sites.

Table S1. Predicted antigenic determinants in Igl-C sequence.

Number	Start Position	End Position	Sequence
1	611	617	KKSCAQL
2	629	658	ISDGFVTCLDCDDSAYIVGSQVGACTQCSP
3	666	674	NKCQLCSTK
4	676	701	SQYGHCAACSATACITCEDINLILTG
5	703	712	KPCTVCKDGF
6	719	736	TDGVYCSPCPAKCKTCKY
7	741	750	KKVECVTCTE
8	757	772	KAPECACPTGTVQLEN
9	774	786	TCQSCSDLSKYPG
10	799	805	TGFIYAT
11	816	822	PYSNCTT
12	837	848	GCAKCDDKCATC
13	850	872	DKDTCLTCADPLKVGSKCDGCKT
14	880	909	ECKPCTNHCSECSSAAECTVCESDTYKVIS
15	912	919	GCNSCVDG
16	926	944	KGTCIPCTSPCTKCVGVKK
17	957	966	KKKIVEECTK
18	972	990	HIAEVPVNGACVCAYGYVE
19	998	1014	KIECQACKAKVNEFCDS
20	1016	1027	NSKDCLRCNAEY
21	1032	1040	GGECVCVEG
22	1045	1061	SWGSCIPCSRHMPHCTK

Table S2. Amino acid sequences of various *Entamoeba* Igls used in the study.

E. histolytica Gal/GalNAc lectin Igl1

GenBank accession no. AAK92361 (1101 aa)

MFILLLFISISLGDYTADKLIGGKEPREAVPHCASVSNGACTSCDTGYELTTTGNNKTCTLKEDMCKTAFSYYDKTNSTNPKCTYCVNGKEVNTSS

HSGNDKCVCKNNVNICESCLLMKDSKCGECIIGMSTTVDGSKLCDNATTEDHAENCVGLLASSTSSKTCDKCFGMYSLQGGKCTQKNDKINKCI

LQVENSCNQCADGYSLSTDKKSCNKFPEHCSKINGNQCLTCMEGYYLSKTDSKCTICTVDNPNNLSEGNECSIYNAEHCTSCNKRCTVSDGVCV

KNHCRLFSPTEENKCTKCDNGYFLTTSGTCSPNLYDGFKTANRTECENGYYLEKDGDKKRCSLCPDPFTECLTSKTPVPGKLNLRSSHLTSTDGPC

KLPGCLLCSDDDTICYKCENGLTLNGTHCYNFDTKSVLGTSGNNHQVCKMRGYDQYEQYLNAFKASDNTYYCPLKDLYLPYYFSVTKGTSDNT

ITIGCVGQLRNVSNDCECNDKHIPTSIDKASDCVSITTKLPSCERTANGNICTQCPVGSHVGKDGKCSCGDAHYFDKDNVCKKCPASCSSCSYDSS

KSKVVCSECYENIQGVTTRNKENECACINDGYKEGPNAEDKKKSCAQLNNNCKKEGKYEISDGFVTCLDCDDSAYIVGSQVGACTQCSPNAFK

DENNKCQLCSTKQSQYGHCAACSATACITCEDINLILTGEKPCTVCKDGFYQIENATDGVYCSPCPAKCKTCKYNTTSKKVECVTCTEQRLKDIK

APECACPTGTVQLENGTCQSCSDLSKYPGCKKTDSCNVDSRTGFIYATECSDGFSGRSPYSNCTTCTKSNYYPKEGEKNGCAKCDDKCATCSDKD

TCLTCADPLKVGSKCDGCKTGYYMSNGECKPCTNHCSECSSAAECTVCESDTYKVISGNGCNSCVDGFYFDEIKGTCIPCTSPCTKCVGVKKDCE

EQETGCNSEKKKIVEECTKCSTKDHIAEVPVNGACVCAYGYVEGTSTEDNKIECQACKAKVNEFCDSCNSKDCLRCNAEYLEAKGGECVCVEG

YYTSSWGSCIPCSRHMPHCTKCTGEGECTTCEDGWKLKDGKCNGAKGIFIMMMIVMLAFMF

E. histolytica Gal/GalNAc lectin Igl2

Lab-identified (1105 aa)

MFILLLFISISLGDYTADKLINNQEPRTAVPHCASVSNGACASCDEGYELKTESGSGSTQKCTLKEETCKSAFSYYDGSDSNSPKCVYCENGKESDT

SSSNNEKCKCKNGVDTCESCLSKDNDKCGECVIGMSTTTNGGQKLCDTVTTDEHAENCVGLTAKDSSSKQCDKCFGMYSLQSGQCTKKNEKIE

KCILQVESSCNQCADGYYINTEKKCTKYPDHCSKMNSDKCNGCMEGYYLNGTECKVCTIDNSKDLSEGNECSIYNAEHCESCNKRCTVSDGVC

VKNHCRLFSPTEENKCTKCDDGYFLTGAGKCSPNLNDGFKTSAKTECQKGYYLEKDGDKKRCSLCPDPFTECLTSQTPVPGKLNLRSAHLTSTDG

PCKLPGCLLCSDDDTICYKCENGLTLNGTHCYNFDVKKVLGTSGNNHQVCKMRGYDQYEQYLNAFKASDNTYYCPLKDLYLPYYFSVTKGSD

NKITIGCVGKDRDVKNDCECNDKYIPKSVDKASDCVSIKTKLPSCERAANENICTQCPVGSHVDSNGKCSCGDAHYFDQNNKCQECPASCSSCSY

DSSKSKVVCSECYENIQGVSTRDKDNECACKKDTPEYKEGLNAEDKKKSCAQLNNNCKEEGHYKISDGFITCLECDDSAYIVDSQTKECAQCAS

NAFKDENNKCQLCSTKKDKYGHCSACSATACIICEDTNLVLAASGSNAQCTVCKDGFYQIESPTDGVYCSPCPAKCKTCKYSADKKEIECVTCTD

QSSVDIKPPTCACLTGTVQLENGTCQSCSDLSKYPGCKTTDTCNVDSRTGYIYATECSDGFSGRSPYSNCTTCIESNYYPKEGEKNGCAKCDDKCA

TCSDKDTCLTCTDPLKIGSKCDECKTGYYMSNGECKPCTNHCSECSSAAECTVCESDTYKVISGNGCNACVDGFYFDEIKGTCIPCTSPCTKCVG

VKKDCEEQETGCNSEKKKIVEECTKCSTKDHIAEVPVNGACVCAYGYVEGTSTEDNKIECQSCKAKVNEFCDSCNSKDCLRCNAEYLEAKGGEC

VCVEGYYTSSWGSCIPCSRHMPHCTKCTGEGECTTCEDGWKLKDGKCNGAKGIFIMMMIVMLAFMF

E. dispar Gal/GalNAc lectin Igl1

GenBank accession no. BAF93871 (1110 aa)

MFIILLFISISLGEYKADKLIKGQEPRTAVPHCASVSNGACTSCDVGYELSSDSSNTQKCTLKQDICKTTFSYYDNSNASSPKCVYCENGKEANTPP

NSNSEKCTCKNGVDNCDTCLSKDSGTKCEECIIGMSTTNTGSSKGCDNATTDDHAENCIGLLASTTSSKTCDKCFGNYTLENGKCTKKNEKISNCI

LQVVNDCNQCADGYYINAEKKCTKYPDHCSKMNGNQCSTCMEGYYLKDSKCNVCTIDNPNNLSEGNECSIYNTEHCTSCNKRCTVSGGFCTKN

HCRLFSLTEENKCAKCDNGYFLTTEGKCSPNLYDGFTTSAKTECQPGYYLEKDGENKRCSLCPDPFTECLTSKTPVPGKLNIKNSHLTSTIGPCKLP

GCLSCSDDDTICYKCENGLTLKGTHCYNTIINDVLGISGKNHKVCKMRGYNQFEQYLNAFKASDNTYYCPLTDLFLPYYFNVTKNSKDMSKITIG

CVGKSRDVKNDCECEAKYIPTSIDKSSDCVSIATKLPSCERAANENICTQCPVGSHVGSDGKCSCGDGHYFDKDNTCKKCPDSCSSCALDSTKNN

VICTSCYENIQGVTTRDKDKKCACVSNDYKEGPNEEDKKKSCAQLNKNCKNEGKYEISDGYVTCLDCDNPAYIVGSQISACTQCSPNAYRNGNE

CVLCSTKQAQFGHCSSCSATACLTCEDNNLILTASGSNVQCTECKDGFYKIENPTDGVYCSPCPAKCKTCKYNTPTKKIECLTCTDTTSQDIKAPEC

ACPKETVQLENGRCKSCSELSKYEGCKTTDTCNVDAKTGYIYATECSENFNGRSPYSNCTACTLSNYYPKNGEKGEGNKNNGCAKCNPECGTCS

DQDICLTCTDSLKVGSKCDRCKTGYYMSNGECKPCTNHCSECTSATECTVCESETYKVISGKGCNSCADGFYFDEIKGACIPCTSPCTKCIGVMK

DCEEGESGCNSEKKKVVEHCTKCSTKDHISEFPLNGACTCAYGYIQNNSTKDNTIECESCKAKVNEFCDSCNSNECLKCNAEYLEVKGEECVCVE

GYYTSSWGSCVPCSRHMAHCTKCSGEGKCTSCEEGWKLEEGNCNGSKGIFIMMVIVMLSFMF

E. dispar Gal/GalNAc lectin Igl2

GenBank accession no. BAF93872 (1106 aa)

MFILLLFISISLGDYKADKLIGDKEPREAVPHCASVSNGACASCDDGYELKTESGSNKCVLKGGTCKSSFSYYDTSNSETPKCTYCEDGKEVDDSS

TSSTDKCTCRNSVSTCDTCLYMGQDNKCGECVIGKYSTTGEKLCDNAIADDPAENCIGLSAKDSSNKACDKCFGSYTLQNNMCSNQLTKIDKCIL

QVENSCNQCADGYYINAEKKCTKYPDHCSKVNGEKCASCMEGYYLTGTECEVCTIDNLDDLSKGDECSIYSAKHCTSCNKRCTVSGEVCVKNH

CRLFSLTDSSKCAKCDSGYFLTSSGTCSPNFYDGFKTSAKEECLPGYYLEKDGENKRCTLCPDPFTECLTSKTPVPGKLNLRSSHLTSNAGPCKLPG

CLSCSDDDTICYKCDEGLTLRGTHCYNLDSVNVLGTSGEEHRVCKMRGYNQFEQYLNAFKASDDTYYCPLTDLFLPYYFSVTKGSSNAITIGCVG

QLRNVSNDCECNDKHVPTSIDKSSDCVSIVTKLPSCERAANENICTQCPVGSHVGSDGKCSCGDGHYFDENNVCQKCPDSCSSCSYDSNKKAVV

CTACYENIQGVTTRDKNSQCACLDERYKEGPNEEDKKKSCAELNENCNQEGKYEISNGYVKCLECNDPAYIVGSEINACSQCSSSAFKDSNNKCQ

LCSTKQDKFGHCSSCSATACITCEDNNLILAASDSNVQCTECKDGFYKIENPTDGVYCSPCPAKCKTCKYDSTKQEVECLTCTDTTSQDIKAPECA

CPTGTVQLENGRCKSCSELSKYEGCKTTDTCNVDAKTGYIYATECSEDFNGRSPYSNCTACTLSNYYPKKGEKGEDGNNNGCAKCGSECATCSD

EHVCLTCAKPLMVGSKCDRCKTGYYMSNGECKPCTNHCSECTSATECTVCESDTYKVISGKGCNSCADGFYFDEIKRACILCTSPCTKCIGVMKY

CEEGESGCNSEKKKVVEHCTKCSTKDHISEFPVNGACTCAYGYIQNNSTKDNTIECESCKAKVNEFCDSCNSNECLKCNAEYLELKGGECVCVE

GYYTTSWGSCIPCSRHMAHCTKCSGEGKCTSCEEGWKLEEGNCNGSKGIFIMMVIVMLSFMF

E. nuttalli Gal/GalNAc lectin Igl1

Lab-identified (1106 aa)

MFILILFISISLGDYTADKLINSQEPRTAVPHCASVSNGACASCDEGYELKTESGSNKKCTLKEETCKSAFSYYDGTTDSNSPKCVYCDNGKESNTP

SGSSTDKCQCKNNINTCDTCLVMGDNSKCDECVIGMSTTVNGGSKLCDTVTTDNAANCVGLTAKDASTKQCDKCFGMYSLENGQCTKKNEKIE

KCILQVESTCNQCADGYYINTDKKCTKYPDHCSKMNGNQCLTCMEGYYLNKADSKCNECTIDNPNNLSEGNECSIYNTEHCTSCNKRCMVSEG

LCVKNHCRLFSPTDDTKCTKCDYGYFLTSAGTCSPNLNDGFTTSAKTECLPGYYLEKDGDKKRCSLCPDPFTECLTSKTAVPGKLNLRSSHLTSTN

GPCKLPGCLLCSDDDTICYKCENGLTLKGAHCYNLDTKQVLGISGKNHQVCKMRGYDQYEQYLNAFKASDNTYYCPLKDLYLPYYFTVTSDSS

KNTIGCVGQHRNVSNDCECNEKYIPTSIDKASDCVSITTKLPSCERAANGNICTQCPIGSHVGKDGKCSCGDAHYFDQNNKCQKCPASCSSCSYD

TSKSKVVCSECYENIQGVTTRDKDNECACKKDTPEYKEGPNEEDKKKSCAQLNGNCKNEGHYEISDGFVTCLDCDDSAYIVDSQTKECAQCAPN

AFKDSSNKCQLCSTKQSQYGHCSACSATACIICEDTNLVLAATGSNAQCTVCKDGFYLIDNPTDGVYCSACPAQCKTCKYDTSKKDVECLSCTDQ

TSSDIKVPECACPTGSVLLESGKCQSCSELSEYEGCKTTDTCNVDSRTGYIYATECSDGFSGRSPYSNCTTCTASNYYPKKGVDTGCAECDAKCAT

CSDKDTCLTCTDPLKVGSKCDGCKTGYYMSNGECVPCTNHCSECSSATECTVCESDTYKVISGNGCNSCVDGFYFDEIKGTCIPCTTPCTKCVGV

KKDCEEQEPGCNSEKKKIVEECTKCSTKDHIAEVPVNGACTCAYGYVEGKSTEDNKIECQACKAKVNEFCDSCNSEECLKCNAEYLEAKGEECV

CVEGYYTSSWGSCIPCSRHMPHCTKCTGEDECTTCEEGWKLQDGKCSGAKGIFIMMMIVMLAFIF

E. nuttalli Gal/GalNAc lectin Igl2

Lab-identified (1103 aa)

MFILILFISISLGDYKADKLINEKEPREAVPHCASVSNGACTSCDTGYQLSPNSENNKKCTLKQEICKTLFSYYDTSNSSNPKCTYCENGKEVNAST

NSGSETCACKNDVTSCETCLVMGDDKKCGECIIGMYATSTNGSGKICDTAVPDDHATSCIGLSATTTSSKTCDKCFGNYTLQDSKCSNLVKKIDKC

ILQVVNDCNQCADGYSLSTDKKKCTAFPEHCSKVNVDKCSGCMEGYYLSKTDSKCNECTIDNPNNLSEGNECSIYNAEHCTSCNKRCMVSNGV

CVKSHCRLFSPTDDTKCTKCDYGYFLTTSGTCSPNLYDGFKTADRTECENGYYLEKDGNKKRCSLCPDPFTECLTSKTAVPGKLNLRSSHLTSTNG

PCKLPGCLLCSDDDTICYKCENGLTLNGTHCYNSVINDVLGTSGKNHKVCKMRGYNQYEQYLNAFKLSNNTYYCPLKDLYLPYYFTVTSDSSNN

SITIGCVGKDRDVKNDCECNDKYVPKSIDKASDCVSIKTKLPFCERAANENICTQCPVGSHVGKDGKCSCGDAHYFDEHNVCKKCPDGCSSCSY

DLNKKNVSCTVCYENIQGVTTRNKDDQCKCVTGYKQGPNEEDKKKSCAQINSNCQKEGNYEISDGFVTCLDCDNPAYIVGSQLSACTQCSPNAF

KDSSNKCQLCSTKTEFGHCSACSATACITCEDTNLVLTGAKPCTECKDGFYKIENVTDGVYCGACPAKCKTCQYNTTSKMVECLSCTEQRLQDIK

APECACPKGTVQLENGTCKSCSELSEYEGCKKTDTCNVDSRTGYIYAMECSDGFSGRSPYSNCTTCTESNYYPKKGEKSGCAICDAKCATCSDKD

TCLTCTDPLKVGSKCDGCKTGYYMSNGKCEPCTNHCSECSSATECTVCESDTYKVISGNGCNSCVDGFYFDEIKGTCIPCTTPCTKCVGVKKDCE

EQEPGCNSEKKKIVEECTKCSTKDHIAEVPVNGACTCAYGYVEGTSTEDNKIECQACKAKVNEFCDSCNSKNCLRCNAEYLEAKGGECVCVEGY

YTSSWGSCIPCSRHMPHCTKCTGEDECTTCEDGWKLKDGKCSGAKGIFIMMMIFMLAFIF

E. moshkovskii presumptive Igl-like sequence

GenBank accession no. NWBR01000099 (5527-8811 bp, reverse; 1094 aa)

MIILYLFISICLAEWIPSNYISGDDPRKTVAYCATAEGGKCTSCDNGFQLVGELCEFKSAANCPNTAYATHYETGNKCSYCTPGEEYDAGCKCKNG

MGTDCKTCVSENTEGVCDACVNGTALSGSGCVGIDSLVHIGSLNEENCIYLKYDGSQVTCDKCFGSKTLSGGACSGDVEKITNCVNQVGSVCST

CKDGYFLNEGNCDAVPGQCLTVTNKVCTKCLEGYVLIGNTCQVCQIDIPSETGDECSIYSTTNCTACNKYCKVDVDSGVCVKNHCDLFGSGGAG

GNEGECVRCENGYFLVGGKCIENMYEGFTDYDKTTCLPGYYLEIEEVEEGIFNKRCSHCVGPFIECISNTVPVPGKINLRITHLTIPTPCENPFCSLCS

DDDSYCFKCDSPGVLVGGRCLLNNAIEVSTTNADKFMACRVRGYDGFDELLSGFNSTGGNIYCIPQKTYLPYYFKVEGSVVTCAGLNRNINNDC

DCNSGFISTSADKTTACSKITLESCEETANNAICLKCPIGAHIISGVEGKGECQCPLTQYPLNGKCVECPPKCISCELDGGNVRCTKCYENNIDILVTR

DLNNLCSCFNSSFVEGPEVSRPYYGCALTNDRCDYYGKDVSYTISEGYWKCSVCDDPAYIPSSAYITYSDCTTCSPSAYLNATGHCVSCSTIQPGCS

VCDGTTCSECLDSNLDKDTNCATCTTTDFYVDPSDKTKCTQCPVKCKGCKYNETEKLVDCIECSSSSTTIVPPLCACGVTSVTLNDGTCKECGNN

PEWANVCDVTVPDNPCGYDYSTGFVHCYKCSDNYKNRVASSNCTSCNSGYFPTEANLATTGGCEKCLDMCTTCNTSTTCITCADKTKQGAYCD

ECRKGYYKQGSDCIKCTNYCSECTSDTECTSCESSIYVVVPGKGCNGCADGYYYDEVRKVCVSCEGGCLKCMGITVDCDPNNDTECQSTGNKT

EHYCKKCEAKNHISEYPVNGECVCEKGYDFYQEEGQSKATCQSCRLKADEFCSSCSNTTCSECNAQYLRIEGGKCVCVDGYYTTSWGSCVPCSR

HMPHCNTCAAKQQCTKCEDGWDLKDGLCSGVVEITILLALLLAFLF

E. invadens presumptive Igl-like sequence

GenBank accession no. AANW03000225 (11227-14448 bp, forward; 1073 aa)

MFLTLFAILLVNADPFLKVGDKEPRETHQNCKKFTRILDKDGNPTNTINCIECENSYFKLEDGKCVYDSTNCNVTDPYVYLSSNECKFCDYGCNS

CDKSGTCTCSFNSDVDTCKQCVSTFDSGSGTKTKKCKYCGQGLATNVNMDACEAAPNGNEVFDKTNCALSGIKEGKQVCLKCFGNKTIDSDKL

HCQYELKVIEGCIRQNGDNCLKCDVGYYLSSKTCVKKDENCNILKETSNDVICTSCREGYYFNDKGKCTMCEVSGEENKQYDSLCELYDANKCI

QCSMRCKVVGSKCVSNHCVEFKSDGKCGICEDGYFALNGECNLFANGGLEMSSGSPTCKAGYYLDYNIETKVYSCKKCKGHFMDCLTEDTPMP

GSSLYDNWKSTKAEPCEDENCAQCADDGVTCYKCAFNNGSPSGVLLNGKCILTPTDNILEIVGTYDTTYKFCKYKGVNSFFEELVPAYNETTNPG

SKCNPILRRSYMYKTQTTGKTEIECAKSNRDVTKNCMCKNGYYQTNASTFTDCTKMSNLEGCIESANGKHCTLCATGAKYTSEGKCVCPKGTY

LKDDKCERCPEKCATCVLETGKTEVTCKTCQPEPYEGQGRDPANKCECKTNFVTEANKIDVCVEKIEGCSTENKYIVQNKQINCLECDKEHMAIG

YGLTQCSQCVDGYYLDEAKDCKVCDHGIGCLTCDKTACTQCKDGNAILGPTSNPTKYCTTCKEGYTFNSTDGTCARCPSVCNGCEIKDEKIECL

GCKNGKQIPNCECENGVYLDPNTNTCVACPNDRKVCKGNCDYGNGIYVHCTECVDVLREPTTNCTTCKDTNRFLDSNGNCEQCAENCNVCTD

KEHCTVCDPATFRTGFTCEKCVKGYYLDTDKCKQCDSKCENCTSATDCEKCIDVNRKPVPGKMCEGCNDGYYLSGESCLTCSKNCKVCEDQTK

CVKCAVENFFKETPVDGTCVCIEGYVYDTKTQTCDPCKDKLNEFCSSCSTEKCSVCNAEYFEPKGGECVCKEGYYTTSWGACVPCDRHISGCVL

CDGKDSCSKCKDGYTLNKTSGKCNGAIKMVIIMVTIALALLF

Table S3. Primers used in the construction of recombinant plasmids.

Primer	Sequence (5' - 3')
**Prokaryotic expression: *E. histolytica* Igl and the segments**	
Igl-14-XhoI-S	CC CTCGAG GATTATACTGCTGATAAGCTC
Igl-294-XhoI-S	CC CTCGAG ACAGAAGAAAATAAATGTACA
Igl-603-XhoI-S	CC CTCGAG GAAGGACCAAATGCAGAAGAT
Igl-749-XhoI-S	CC CTCGAG ACTGAACAAAGGCTAAAAGAT
Igl-871-XhoI-S	CC CTCGAG AAAACAGGTTATTATATGTCA
Igl-989-XhoI-S	CC CTCGAG GTTGAAGGCACTTCTACAGAA
Igl-382-XhoI-AS	CC CTCGAG TTA AAGTTTGCATGGTCCATC
Igl-753-XhoI-AS	CC CTCGAG TTA TAGCCTTTGTTCAGTGCA
Igl-873-XhoI-AS	CC CTCGAG TTA ACCTGTTTTACATCCATC
Igl-991-XhoI-AS	CC CTCGAG TTA GCCTTCAACATATCCATA
Igl-1088-XhoI-AS	CC CTCGAG TTA AATGCCTTTAGCTCCATT
**Eukaryotic expression: *E. histolytica* EGFP-Igl-C3**	
SP-NheI-S1	CG GCTAGC CACCATGGGAATCCAAGGAGGGTCTGTCCTGTTC
SP-AS1	GAAGACAGCCAGGACGAGCAGCAGCCCGAACAGGACAGACCC
SP-S2	CGTCCTGGCTGTCTTCTGCCATTCAGGTCATAGC
SP-XhoI-AS2	CC CTCGAG GCTATGACCTGAATGGCAGAAG
Linker-BamHI-S	CG GGATCC C GGTGGCGGTGGCTCGGGCGGTGGTGGCTCC GGTGG
	CGGAGGGTCTATGGT
EGFP-S	CGGAGGGTCTATGGTGAGCAAGGGCGAGGA
EGFP-AS	GTTACACAGGTC CTTGTACAGCTCGTCCAT
GPI-S1	ACGAGCTGTACAAG GACCTGTGTAACTTTAACGAACAGCTTGAAAATGGTGGGA
GPI-AS1	GAGTCACCAGCAGAAGAACTGTTTTCTCTGATAAGGATGTCCCACCATTTTCA
GPI-S2	TTCTGCTGGTGACTCCATTTCTGGCAGCAGCCTGGAGCCTTCATCCCTGA
GPI-HindIII-AS2	CC AAGCTT GG TCAGGGATGAAGGCTCCAGGCT
Igl-989-XhoI-S	CC CTCGAG GTTGAAGGCACTTCTACAGAA
Igl-1088-EcoRI-AS	CG GAATTC AATGCCTTTAGCTCCATTACA
**Eukaryotic expression: *E. histolytica* Igl-C**	
Igl-603-SgfI-S	CC GCGATCGCC ATG GAAGGACCAAATGCAGAA

Igl-1088-Linker-AS	AGATCCTCCTCCGCC AATGCCTTTAGCTCCATT
FLAG-S	GGCGGAGGAGGATCT GATTACAAGGACGATGATGACA
FLAG-MluI-AS	CG ACGCGT TAA CTTGTCATCATCGTCCTTGTAATC
**Point mutations**	
Igl-mutation-636-S	GATGGATTTGTTACCGGTCTTGACTGTGATG
Igl-mutation-636-AS	CATCACAGTCAAGACCGGTAACAAATCCATC
Igl-mutation-854-S	TCAGATAAAGACACTGGTTTAACATGTGCTG
Igl-mutation-854-AS	CAGCACATGTTAAACCAGTGTCTTTATCTGA
Igl-mutation-913-S	ATTAGTGGAAATGGAGGTAATTCATGTGTAG
Igl-mutation-913-AS	CTACACATGAATTACCTCCATTTCCACTAAT
Igl-mutation-1035-S	GCAAAAGGAGGAGAAGGTGTATGTGTTGAAGGA
Igl-mutation-1035-AS	CTTCAACACATACACCTTCTCCTCCTTTTGC
Igl-mutation-1049-S	TCTAGTTGGGGATCAGGTATTCCATGTTCAA
Igl-mutation-1049-AS	TTGAACATGGAATACCTGATCCCCAACTAGA
Igl-mutation-1068-S	ACAGGAGAAGGCGAAGGCACAACATGTGAAG
Igl-mutation-1068-AS	CTTCACATGTTGTGCCTTCGCCTTCTCCTGT

All primers were synthesized by Invitrogen company.

Table S4. Primers used in quantitative real-time RT-PCR.

Amplified gene		Primer sequence (5' - 3')
IL-1β	S	ACATCAGCACCTCACAAGCAG
	AS	TTAGAAACAGTCCAGCCCATAC
IL-6	S	TGCCTTCTTGGGACTGAT
	AS	TTGCCATTGCACAACTCTT
TNF-α	S	GTCGTAGCAAACCACCAA
	AS	GGCAGCCTTGTCCCTTGA
iNOS	S	TCCTGGAGGAAGTGGGCCGAAG
	AS	CCTCCACGGGCCCGGTACTC
GAPDH	S	AGGTCGGTGTGAACGGATTT
	AS	ACTGTGCCGTTGAATTTGCC

All primers were synthesized by Invitrogen company.
